# The association between constipation and stroke based on the NHANES and Mendelian randomization study

**DOI:** 10.3389/fnins.2023.1276032

**Published:** 2023-11-07

**Authors:** Wenjuan Du, Shengkai Yang, Hai Zhou, Yueju Wu, Yong Cai, Hai Meng, Hongwei Teng, Ai Feng He

**Affiliations:** Binhai County People's Hospital, Yancheng, China

**Keywords:** constipation, stroke, large-artery atherosclerotic stroke, ischemic stroke, Mendelian randomization analysis

## Abstract

**Background:**

This study aimed to investigate putative causal effects between constipation and stroke using bidirectional Mendelian randomization (MR) analysis.

**Methods:**

Based on the cross-sectional study, logistic regression models were developed to assess the association between constipation and stroke prevalence. Subsequently, genome-wide association studies statistics were employed to perform MR analysis between constipation and stroke, as well as its subtypes. The inverse variance weighting (IVW) method was the primary method, complemented by four additional methods, namely weighted median, weighted mode, simple mode, and MR-Egger regression. Cochran’s Q test, MR-Egger intercept test, MR Pleiotropy RESidual Sum and Outlier, and MR Steiger test were performed to assess heterogeneity and pleiotropy effects.

**Results:**

Constipation was associated with a greater risk of stroke even after adjusting for all covariates in logistic regression [odds ratio (OR) = 1.46, 95% confidence interval (CI) = 1.01–2.09, *p* = 0.042]. IVW MR analysis revealed that constipation affected large artery atherosclerosis (LAS; IVW OR = 1.5, 95% CI = 1.07–2.104, *p* = 0.019). No significant or suggestive association was observed with the risk of stroke or its various subtypes in MR analysis. Meanwhile, reverse MR analysis revealed no significant causal relationship between stroke or other stroke subtypes and constipation. The results of sensitivity analyses revealed no significant horizontal pleiotropy affecting causal estimates.

**Conclusion:**

While cross-sectional studies have established that constipation increases the risk of stroke, this two-sample bidirectional MR analysis revealed a positive correlation between constipation and LAS. However, no such correlation was observed between constipation and stroke, including its various subtypes.

## 1. Introduction

Stroke represents a prevalent global cause of mortality and disability (GBD 2017 DALYs and HALE Collaborators, 2018). Over the past few decades, improvements in recanalization techniques and primary and secondary prevention have led to a reduction in stroke prevalence and mortality (GBD 2019 Stroke Collaborators, 2021). However, the concerning trend of stroke occurring at younger ages underscores the importance of stroke prevention and treatment strategies for all adults (Danaei et al., 2011). This is particularly crucial as the aging global population and increased life expectancy project higher absolute mortality rates among patients with stroke in the future (Foerch et al., 2008). Rapid action is required to identify risk factors for stroke for early prevention and intervention. Furthermore, patients with stroke experience various non-neurological complications, including gastrointestinal diseases, bacterial infections, and immune system disorders during their recovery phases (2021). These complications not only place substantial burdens on the overall health of the patient but also impede the process of brain recovery after a stroke. Therefore, prompt risk assessment and preventive management of gastrointestinal diseases in patients after brain injury could mitigate systemic inflammation and improve disease prognosis. Notably, several studies have suggested a potential link between constipation and stroke (Sumida et al., 2019; Sundbøll et al., 2020; Dong et al., 2023).

In daily clinical practice, constipation emerges as one of the most common symptoms of gastrointestinal diseases, signifying a global health concern (Bassotti, 2013; Tomita et al., 2021). The prevalence of constipation ranges from 3% to 79% across adult populations, dependent on factors such as age, gender, and the definition of constipation (Gosmanova et al., 2014). In another cohort of 3,359,653 veterans from the United States, patients with constipation exhibited a 19% higher likelihood of experiencing ischemic stroke (IS) compared to those without constipation (Sumida et al., 2019). Furthermore, research indicates a statistically significant association between constipation and overall mortality from cardiovascular diseases (Honkura et al., 2016). Courtney et al. analyzed the relationship between constipation and stroke and transient ischemic attack. After adjusting for confounding factors, constipation was associated with an increased risk of cardiovascular events among hospitalized patients aged ≥ 60 years (Judkins et al., 2023). These findings suggest that interventions targeting constipation might reduce cardiovascular risk among older individuals.

Nowadays, Mendelian randomization (MR) analysis plays an important role in evaluating potential causal relationships between various exposures and clinical outcomes. Unlike observational associations, genetic associations remain impervious to classical confounding or reverse causation, given that genes are randomly allocated at conception. Moreover, the random isolation and independent classification of genetic polymorphisms enables MR analysis to minimize the influence of confounders by employing genetic markers as instrumental variables (IVs) for the exposures of interest (Burgess et al., 2021). In this study, the association between constipation and stroke was demonstrated using logistic regression analysis, Subsequently, this association was reinforced by conducting a two-sample bidirectional MR analysis using large-scale genome-wide association study (GWAS) data to further confirm the association.

## 2. Materials and methods

### 2.1. Cross-sectional study

#### 2.1.1. Study population

**Figure 1:**
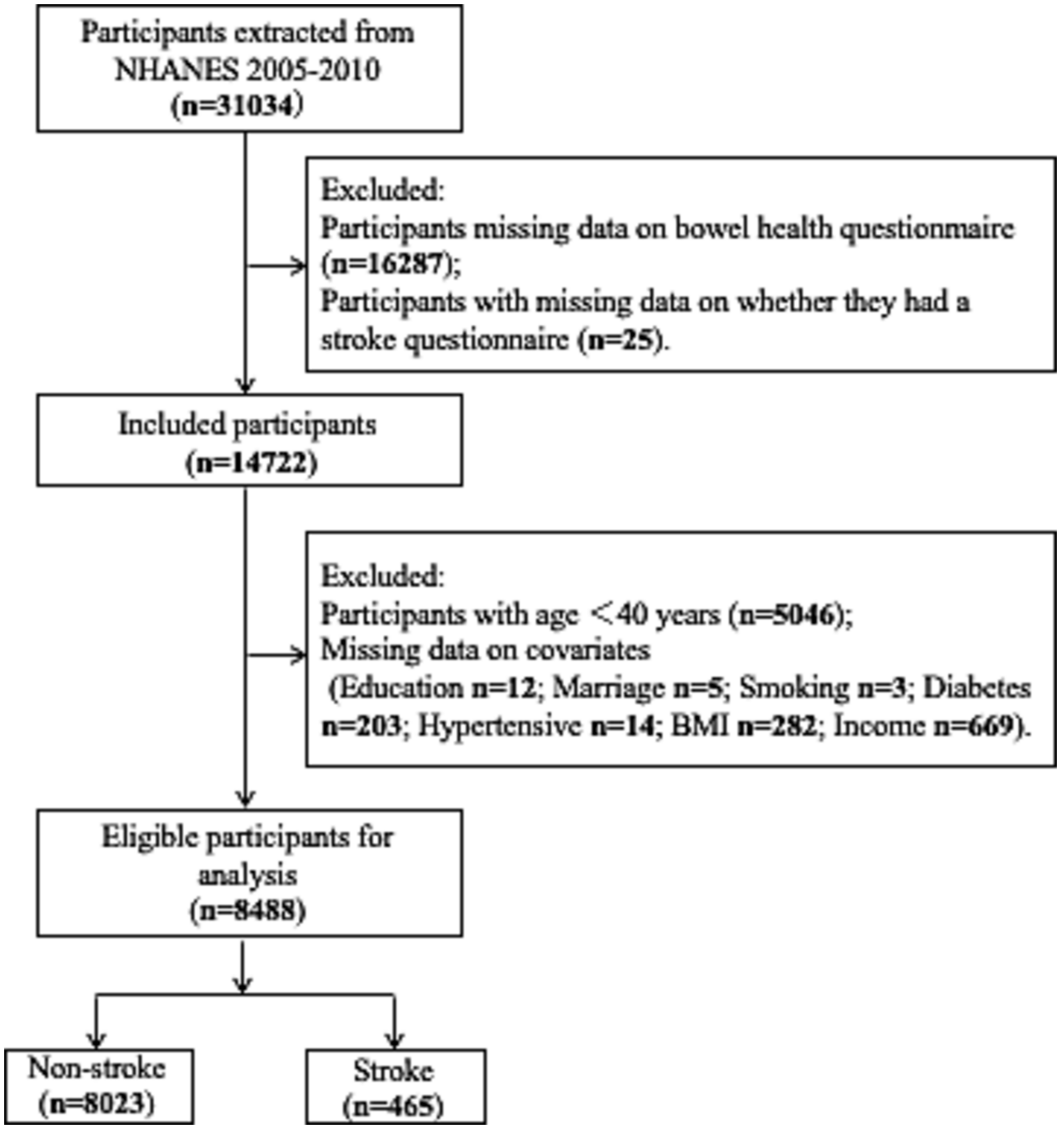
Flow chart of the enrolled patients.

The data examined were sourced from the National Health and Nutrition Examination Survey (NHANES) for three time intervals: 2005–2006, 2007–2008, and 2009–2010, resulting in a sample size of 31,034 individuals. Individuals aged < 40 years with no gut-related or stroke data were excluded (*n* = 21,358) according to the age of stroke onset. Furthermore, individuals with missing data on covariates, including education, marital status, income, smoking, diabetes, and hypertension, were also excluded (*n* = 1,188) from the study. Finally, 8,488 individuals were included in the study (Figure 1).

All participants in the survey provided written informed consent, and the research conducted as part of NHANES adhered to the ethical standards established by the National Centre for Health Statistics Research Ethics Review Board^1^ for approval. All procedures were conducted in accordance with the established standard guidelines.^2^

#### 2.1.2. Exposure: definition of constipation

In this study, based on the NHANES database, constipation was defined according to the characteristics of bowel movements or the frequency of bowel movements (Markland et al., 2013; Wilson, 2020). This was done primarily by collecting 30-day records of stool traits and defecation frequency.

Stool traits were assessed using the Bristol stool form scale, comprising a range of colored cards and detailed descriptions for each of the seven stool types, which were as follows: (1) separate hard lumps, like nuts; (2) sausage-like, but lumpy; (3) like a sausage but with cracks in the surface; (4) smooth and soft, like a sausage or snake; (5): soft blobs with clear-cut edges; (6): fluffy pieces with ragged edges, indicative of a mushy stool; (7): watery consistency with no solid pieces. Study participants were required to identify their stool type by comparing it to the provided photographs. Types 1 or 2 were categorized as constipated, while types 3–7 were classified as non-constipated (Li et al., 2021).

Constipation was also defined in cases when the frequency of bowel movements was less than thrice a week, with individuals reporting three or more times per week being categorized as non-constipated (Liu et al., 2022).

#### 2.1.3. Outcome: definition of stroke

Stroke history was determined through patient self-report, with data collected via a structured questionnaire administered by trained professionals within the patient’s home environment.

This study focused on IS, which has further been categorized into various subtypes, including lacunar stroke (LS), large-artery atherosclerotic stroke (LAS), small-vessel stroke (SVS), and cardioembolic stroke (CES).

#### 2.1.4. Covariates

In the past decades, the etiology and risk factors of stroke have been intensively studied. Potential factors related to stroke, known as covariates, include age, gender, race, family income, education level, and obesity, which are associated with stroke occurrence. To control the influence of the above-mentioned confounding factors on the results of the study, the statistical models in this study were adjusted for covariates to control the influence of the above-mentioned confounding factors on the results, thereby reducing the confounding bias they might introduce. The covariates adjusted were as follows: (a) demographic characteristics: age was divided into two categories (40–59 years, ≥60 years); (b) race was classified into five categories (Mexican American, non-Hispanic white, non-Hispanic black, other Hispanic, and other races, which included multiracial groups); (c) gender (male and female); (d) marital status (married, never married, others, including divorced, widowed, etc.); (e) education level (below high school level, high school level, and above); (f) poverty–income ratio (PIR) was classified into three categories (low income <1, middle income 1–3, and high income ≥ 3; Chen et al., 2019); (g) smoking status (never smoked, former smoker, and current smoker; Gunzerath et al., 2004); (h) drinking status was classified based on the alcohol intake, as never drinkers, moderate drinkers (1–2 drinks per day for men and 1 drink per day for women), heavy drinkers (more than 2 drinks per day for men or 1 drink per day for women), and unknown drinkers; (i) body mass index (BMI; normal: <25 kg/m^2^, overweight: 25 to <30 kg/m^2^, and obese: ≥30 kg/m^2^); (j) and underlying diseases (self-reported doctor-diagnosed hypertension or diabetes and current use of antihypertensive or glucose-lowering drugs were considered as indicators of hypertension or diabetes). These standardized interviews and questionnaires were administered by trained medical professionals.

#### 2.1.5. Establishment of the study model

Four logistic regression models were constructed to assess the association between constipation and stroke risk (Liu et al., 2022). The first model offered a preliminary estimate of the association without adjusting for any covariates. The second model was adjusted for age, gender, race, and education. The third model extended the adjustments to include marital status, BMI, PIR, hypertension, and diabetes. The fourth model introduced additional adjustments for smoking and alcohol consumption.

#### 2.1.6. Statistical analysis

All analyses were performed using R software (version 4.2.3) and Stata 17 (United States). Categorical variables are presented as weighted percentages with 95% confidence intervals (CIs). The chi-square test and *t*-test were used for between-group comparisons. Statistical significance was set at *p* < 0.05.

### 2.2. MR study

#### 2.2.1. Study design

**Figure 2:**
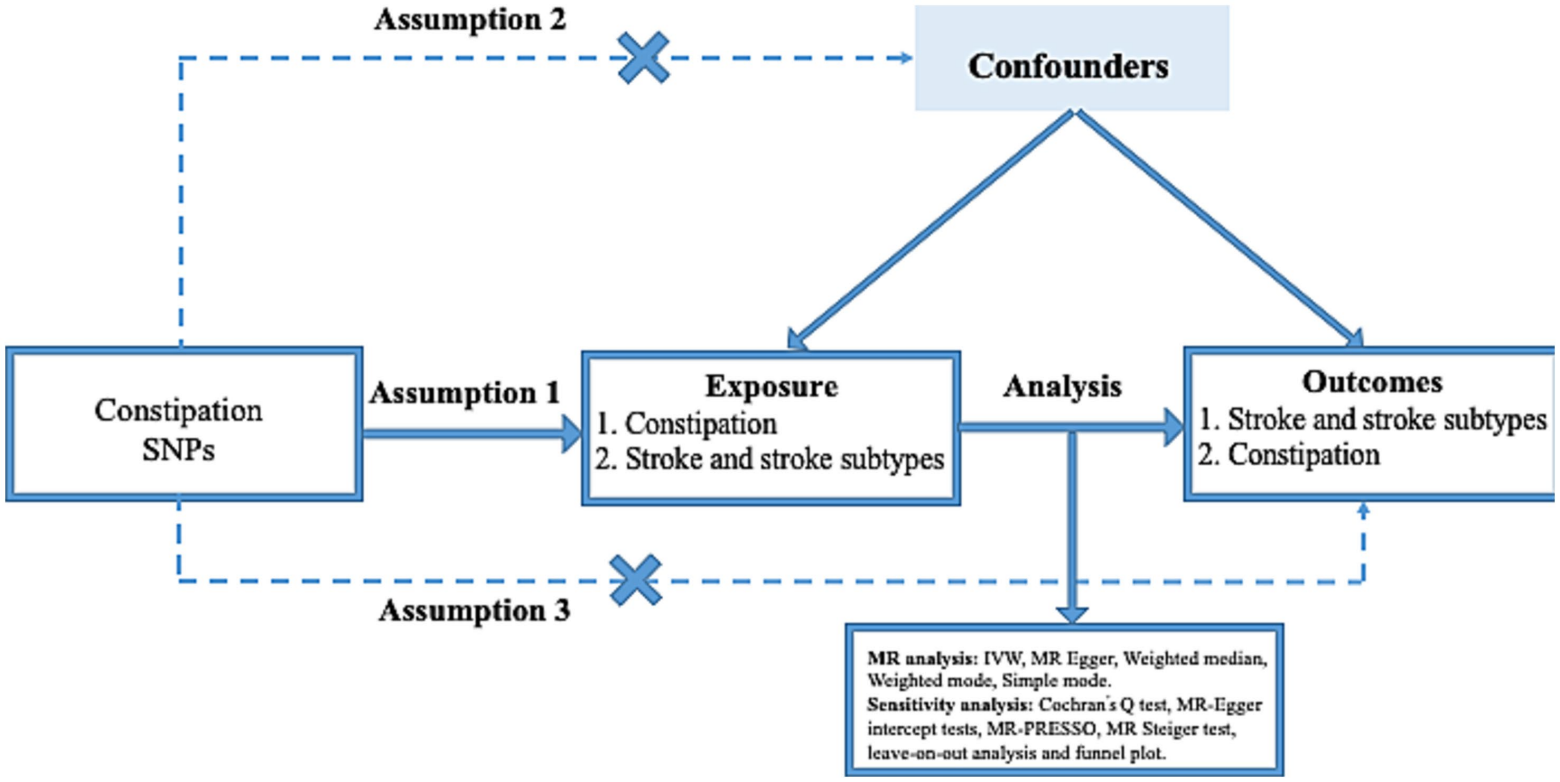
Three key assumptions of the Mendelian randomization study. (1) Genetic variants are significantly associated with exposure constipation; (2) the genetic variants remain unaffected by potential confounding factors; (3) the genetic variants can exert their influence on stroke only through constipation, without any direct effects. SNP, single nucleotide polymorphism.

Figure 2 presents the overall design flow of this MR. Three hypotheses underpin this analysis: (a) genetic variants are significantly associated with exposure constipation; (b) the genetic variant remains unaffected by potential confounding factors; (c) the genetic variants can exert their influence on stroke only through constipation, without any direct effects (Burgess et al., 2015; Evans and Davey Smith, 2015).

#### 2.2.2. Data sources

The public database^3^ and the Finland GWAS data^4^ were used. The total study of constipation cohort was derived from FinnGen’s R8 series and comprised 342,499 individuals, including 30,490 cases and 312,009 controls. In our analysis of stroke-related data, data was drawn from the MEGASTROKE Consortium, which included 446,696 participants of European descent, comprising 40,585 stroke cases and 406,111 controls (Malik et al., 2018). Among these stroke cases, 34,217 participants were categorized as having experienced IS and were further divided into LS (6,030 cases), LAS (4,373 cases), SVS (5,386 cases), and CES (7,193 cases; Traylor et al., 2021). No overlapping populations existed between the exposure and outcome GWAS datasets.

#### 2.2.3. Selection of IVs for constipation

First, an initial screening was conducted for eligible single nucleotide polymorphisms (SNPs; *p* < 5 × 10^−8^). However, considering the limited number of SNPs obtained, this threshold was adjusted to *p* < 5 × 10^−6^. Second, a process was implemented to eliminate SNPs associated with other SNPs or exhibited higher *p*-values, with a criterion of *R^2^* > 0.001 (aggregation window 10,000 kb). Third, the strength of individual SNPs was assessed by calculating the F-statistic, with a threshold of F-statistic greater than 10, which is currently recognized as indicative of the SNP’s capacity to sufficiently mitigate potential bias (Palmer et al., 2012). Fourth, SNPs associated with the outcome by conducting separate searches in PhenoScanner to identify potential confounders associated with stroke were excluded. Fifth, SNPs that were subsequently associated with the results were further eliminated using MR Pleiotropy RESidual Sum and Outlier (MR-PRESSO). Finally, the SNPs associated with constipation were identified and retained as IVs.

#### 2.2.4. Statistical analysis

In this MR analysis, five methods, namely MR-Egger, weighted median, simple mode, weighted mode, and inverse-variance weighting (IVW), were employed, among which IVW served as the primary method. MR-Egger not only facilitates MR analysis but also serves as a tool for assessing pleiotropy. Sensitivity analyses were conducted, which encompassed assessments for heterogeneity and pleiotropy. Heterogeneity testing was aimed at evaluating the difference between each IV and was determined through Cochran’s Q test, where a *p*-value below 0.05 indicated the presence of heterogeneity, while a *p*-value exceeding 0.05 signaled its absence. The pleiotropy test aimed to validate the reliability of the MR analysis results, which is commonly expressed by the intercept term of MR-Egger’s method; an intercept value of >0.05 indicated an absence of horizontal pleiotropy. If pleiotropy was detected, the MR analysis results were considered not reliable. Furthermore, the MR-PRESSO test was performed, which had three key components: (a) the identification of horizontal pleiotropy; (b) the correction of horizontal pleiotropy via outlier removal; (c) testing of significant differences in the causal estimates before and after the correction for outliers. Additional sensitivity analysis was performed using the leave-one-out plot and funnel plot. All statistical analyses were performed using the “TwoSampleMR” and “MR-PRESSO” packages in R version 4.2.3. Statistical significance was set at *p* < 0.05.

## 3. Results

### 3.1. Cross-sectional study

#### 3.1.1. Characteristics of the study participants

A total of 8,488 individuals were included in the study. Table 1 compares the baseline characteristics of the stroke and non-stroke groups. The overall stroke prevalence was 5.4%, with 12.67% of the patients with stroke presenting constipation, compared to 8.54% of the patients without stroke (*p* = 0.009). Additionally, the influence of age, race, gender, marital status, education level, family income, hypertension, and diabetes on stroke incidence was analyzed. Age demonstrated a significant statistical difference, with a higher age correlating with an elevated stroke risk. Although the incidence of stroke was 43.02% among males and 56.97% among females, it was not statistically significant. Among various ethnic groups, non-Hispanic whites exhibited the highest proportion of stroke cases (75.76%), while other Hispanic groups exhibited the lowest proportion (1.69%). An analysis of education level revealed that the stroke rate was 43.84% among those with education beyond the university level. Regarding hypertension and diabetes, the stroke incidence was significantly higher among individuals with hypertension, accounting for 77.55%, and among those with diabetes, constituting 32.25% of the cases. Our analysis found that stroke was more likely to occur among older individuals, non-Hispanic whites, married individuals, those with higher education levels, and individuals with diabetes or hypertension.

**Table 1:** Weighted characteristics of overall participants based on stroke grouping, National Health and Nutrition Examination Survey 2005–2010.

Characteristic	Total (%)	Stroke	Stroke	Value of *p*
		No (%)	Yes (%)	
Total	8,488	8,023 (94.5)	465 (5.4)	
Age				<0.001
40–59 years	62.99 (61.76–64.21)	64.41 (63.16–65.63)	30.20 (24.92–36.06)	
≥60 years	37.01 (35.79–38.24)	35.58 (34.36–36.83)	69.79 (63.93–75.07)	
Gender				0.095
Male	47.70 (46.35–49.06)	47.90 (46.51–49.29)	43.02 (37.62–48.59)	
Female	52.30 (50.94–53.65)	52.09 (50.70–53.48)	56.97 (51.40–62.37)	
Race				<0.001
Mexican	5.38 (5.05–5.73)	5.45 (5.11–5.81)	3.69 (2.58–5.25)	
**American**				
Other Hispanic	3.03 (2.72–3.38)	3.09 (2.76–3.45)	1.69 (0.83–3.38)	
Non-Hispanic	77.34 (76.41–78.24)	77.40 (76.44–78.33)	75.76 (71.47–79.60)	
**White**				
Non-Hispanic	10.03 (9.51–10.58)	9.83 (9.30–10.38)	14.63 (11.96–17.79)	
**Black**				
Other Race^a^	4.22 (3.66–4.85)	4.21 (3.65–4.86)	4.20 (2.31–7.52)	
Educational level				<0.001
Below high school	17.89 (17.01–18.80)	17.48 (16.59–18.41)	27.23 (22.90–32.04)	
High school	25.33 (24.16–26.53)	25.17 (23.97–26.40)	28.91 (24.04–34.33)	
Above high school	56.78 (55.46–58.10)	57.34 (55.98–58.69)	43.84 (38.28–49.57)	
Marital status				<0.001
Married	64.91 (63.62–66.16)	65.36 (64.06–66.64)	54.37 (48.76–59.87)	
Never married	5.92 (5.34–6.56)	5.96 (5.36–6.62)	4.80 (3.03–7.51)	
Widowed/divorced/separated	29.17 (28.00–30.38)	28.66 (27.47–29.89)	40.82 (35.46–46.41)	
Poverty–income ratio				<0.001
<1	9.49 (8.89–10.12)	9.22 (8.61–9.86)	15.57 (12.31–19.51)	
1–3	34.20 (32.99–35.43)	33.45 (32.21–34.71)	51.46 (45.83–57.06)	
>3	56.32 (55.00–57.62)	57.32 (55.98–58.65)	32.95 (27.63–38.75)	
Constipation				0.009
No	91.37 (90.59–92.09)	91.54 (90.75–92.27)	87.32 (83.22–90.54)	
Yes	8.63 (7.91–9.40)	8.45 (7.72–9.24)	12.67 (9.45–16.77)	
Smoking status				0.008
Never	50.07 (48.71–51.43)	50.45 (49.06–51.84)	41.23 (35.77–46.93)	
Former	30.93 (29.71–32.18)	30.65 (29.40–31.94)	37.38 (32.20–42.87)	
Current	18.98 (17.95–20.06)	18.88 (17.82–19.99)	21.37 (17.11–26.35)	
Alcohol consumption				<0.001
Non-drinkers	10.72 (9.98–11.52)	10.46 (9.71–11.27)	16.79 (13.10–21.26)	
Moderate-drinkers	38.36 (37.04–39.71)	38.83 (37.47–40.22)	27.55 (22.74–32.95)	
Heavy drinkers	32.46 (31.18–33.76)	32.90 (31.58–34.24)	22.26 (17.92–27.29)	
Other^b^	18.43 (17.46–19.45)	17.79 (16.80–18.82)	33.38 (28.31–38.87)	
Diabetes				<0.001
No	87.90 (87.08–88.67)	88.77 (87.95–89.53)	67.74 (62.39–72.65)	
Yes	12.09 (11.32–12.91)	11.22 (10.46–12.042)	32.25 (27.34–37.60)	
Hypertension				<0.001
No	58.50 (57.17–59.81)	60.05 (58.70–61.39)	22.44 (17.89–27.75)	
Yes	41.49 (40.18–42.82)	39.94 (38.60–41.29)	77.55 (72.24–82.10)	
Body mass index				0.086
<25 kg/m^2^	24.94 (23.76–26.15)	25.12 (23.92–26.37)	20.60 (16.35–25.62)	
25–29.9 kg/m^2^	31.53 (30.28–32.80)	31.59 (30.31–32.90)	30.01 (25.12–35.41)	
≥30 kg/m^2^	43.52 (42.18–44.87)	43.27 (41.89–44.65)	49.37 (43.78–54.98)	

Values are weighted as means ± standard error or weighted % (95% confidence interval). *p*-values are weighted. *p*-values were tested using the chi-square test or Fisher’s exact test. ^a^Other races comprise multi-racial individuals. Other information loss. ^b^Percentages might not sum up to 100 due to rounding of values.

#### 3.1.2. Association between constipation and risk of stroke

Table 2 illustrates the association between constipation and the risk of stroke. In model 1 (unadjusted), constipation was associated with an increased risk of stroke [odds ratio (OR) = 1.57, 95% CI = 1.12–2.22, *p* = 0.01]. After adjusting for gender, age, race, and education level in model 2, the association between constipation and stroke decreased (OR = 1.51, 95% CI = 1.07–2.15, *p* = 0.02). Further adjustments in model 3, which included marital status, BMI, PIR, hypertension, and diabetes resulted in a continued reduction in the impact of constipation on stroke risk (OR = 1.48, 95% CI = 1.03–2.13, *p* = 0.033). In model 4, after adjusting for smoking and drinking status based on model 3, the correlation remained stable (OR = 1.46, 95% CI = 1.01–2.09, *p* = 0.042).

**Table 2:** Odds ratio (95% confidence interval) between constipation and the weighted stroke risk for overall participants, National Health and Nutrition Examination Survey 2005–2010.

	Model 1	Model 2	Model 3	Model 4
	OR (95% CI), *p*	OR (95% CI), *p*	OR (95% CI), *p*	OR (95% CI), *p*
Constipation				
No	Reference	Reference	Reference	Reference
Yes	1.57 (1.12–2.22) 0.010	1.51 (1.07–2.15) 0.020	1.48 (1.03–2.13) 0.033	1.46 (1.01–2.09) 0.042

Model 1: Non-adjusted model. Model 2 adjusted for gender, age (years), race, and educational level (less than high school, high school, or above high school). Model 3 adjusted for factors included in model 2 along with additional adjustment for marital status (married, widowed, divorced, separated, never married, or living with a partner), poverty–income ratio (<1, 1–3, >3), hypertension (yes or no), diabetes (yes or no), and body mass index (<25 kg/m^2^, 25–29.9 kg/m^2^, or ≥ 30 kg/m^2^). Model 4 adjusted for factors included in model 2 along with additional adjustment for smoking status (never, former, or current) and alcohol consumption (non-drinkers, moderate-drinkers, or heavy drinkers). OR, odds ratio; 95% CI, 95% confidence interval. The bold values indicate statistically significant values of *p* < 0.05.

**Figure 3:**
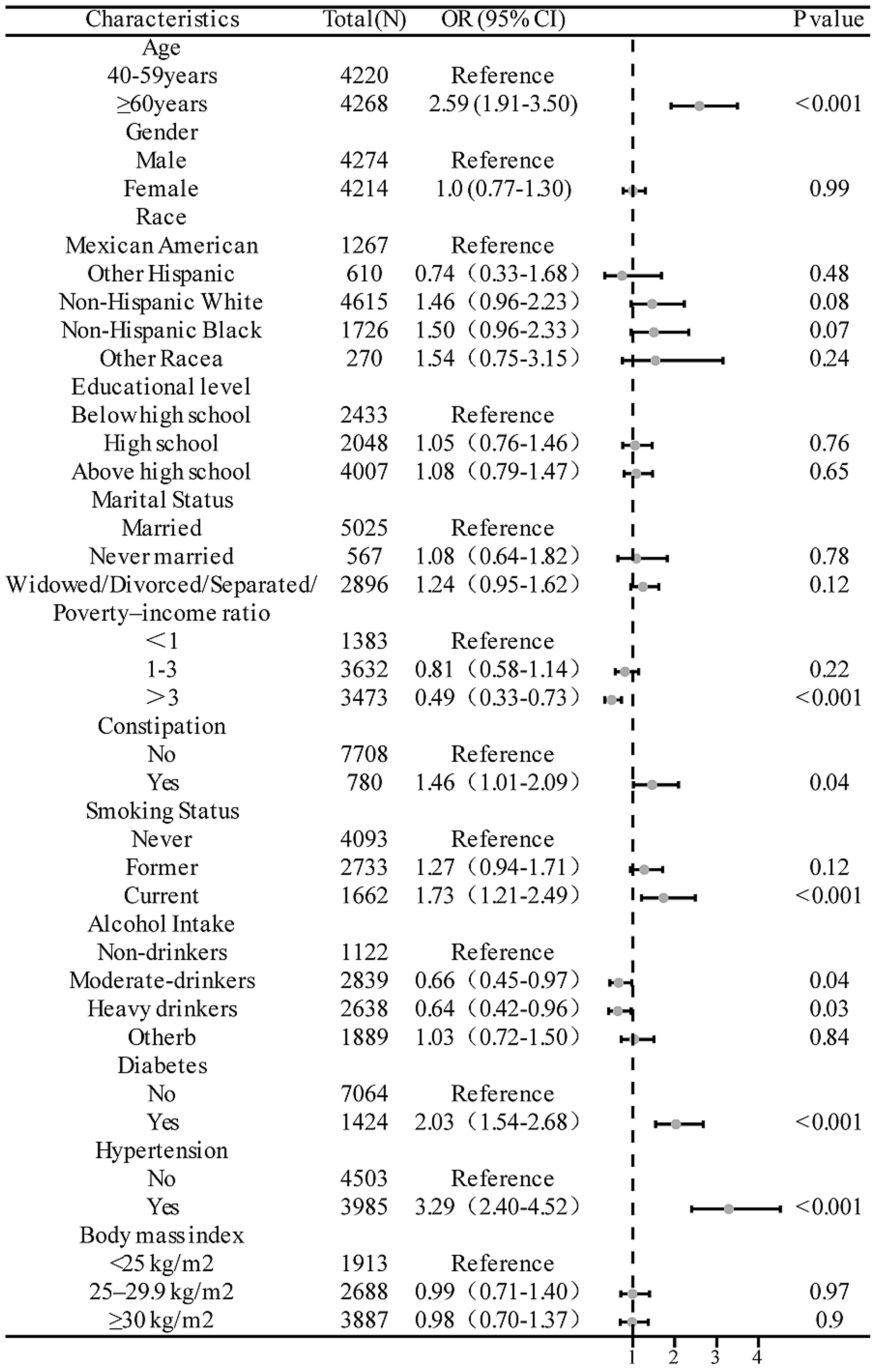
Forest plots of the odds ratios between constipation and stroke risk adjusted for all confounding factors.

Figure 3 presents the results from the analysis of the overall population after adjustment for all confounders, as per model 4. These findings revealed that constipation was associated with an increased risk of stroke (OR = 1.46, 95% CI = 1.01–2.09, *p* = 0.042). The risk of stroke exhibited a positive correlation with age, with individuals aged over 60 years experiencing a 1.59-fold increase in risk compared to those aged 40–59 years old (OR = 2.59, 95% CI = 1.91–3.50, *p* < 0.001). Gender did not impact stroke risk (OR = 1.0, 95% CI = 0.77–1.30, *p* = 0.99). Furthermore, individuals with higher income levels displayed a significantly lower risk of stroke compared to those with lower income (OR = 0.49, 95% CI = 0.33–0.73, *p* < 0.001). Current smokers were at a significantly higher risk compared to those who had never smoked (OR = 1.73, 95% CI = 1.21–2.49, *p* < 0.001). Alcohol consumers exhibited a reduced risk of stroke compared with non-drinkers (OR = 0.64, 95% CI = 0.42–0.96, *p* = 0.03). Additionally, individuals with diabetes had a 2.03-fold greater risk of stroke compared to those without diabetes (OR = 2.03, 95% CI = 1.54–2.68, *p* < 0.001). Similarly, individuals with hypertension faced a significantly higher risk of stroke compared to those without hypertension (OR = 3.29, 95% CI = 2.40–4.52, *p* < 0.001).

### 3.2. MR study

#### 3.2.1. Selection of genetic IVs for MR (constipation to stroke)

In this study, 18 constipation-associated SNPs that met the currently acceptable genome-wide significance thresholds (*p* < 5 × 10^−6^, *R^2^* < 0.001, kb = 10,000) for exposure were identified (Supplementary Table S1). The calculation of F-statistics, which are robust tools used in our MR analyses, indicated the strength of these SNPs (all F-statistics > 10; Supplementary Table S1). Outliers identified through the MR-PRESSO analysis were excluded, and the SNPs that remained after excluding ambiguous and palindromic SNPs were retained as IVs. Detailed information regarding the IVs for each type of stroke and its associated subtype is provided in Supplementary Tables S2–S7.

#### 3.2.2. Causal effects of constipation on stroke and its subtypes

**Figure 4:**
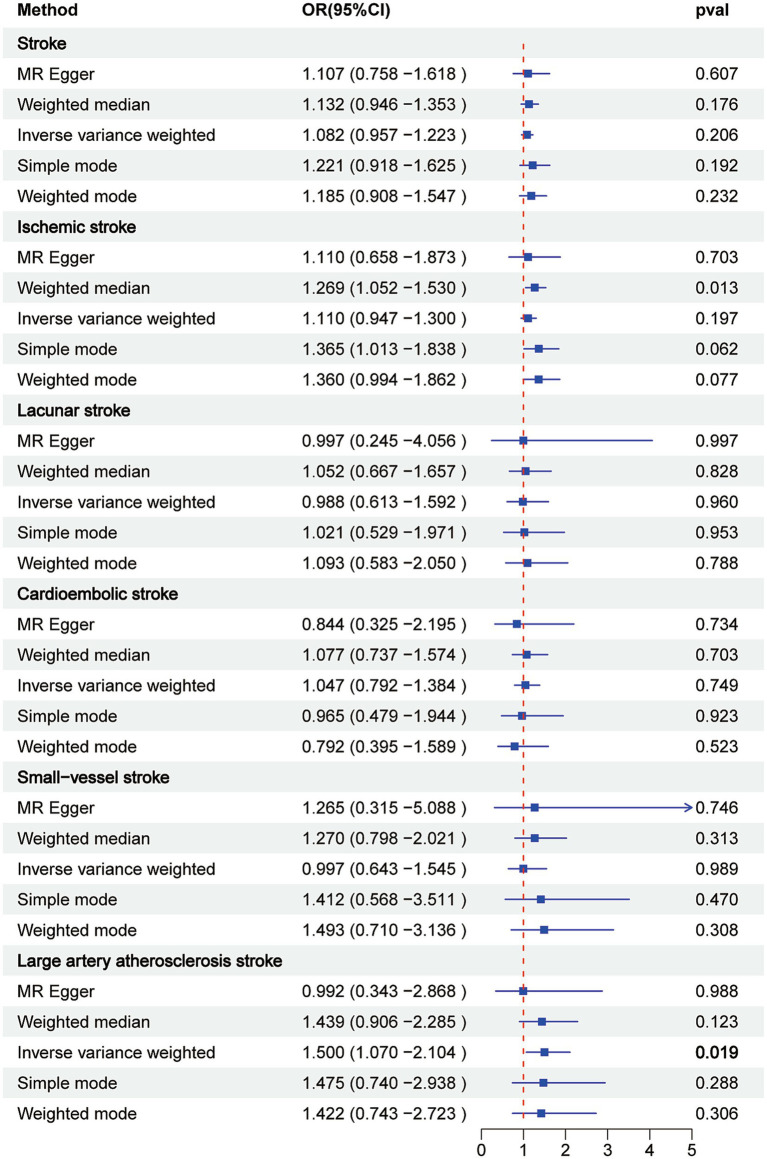
Associations of genetically predicted constipation with stroke and its subtypes by Mendelian randomization analysis.

IVW analysis highlighted a significant association between constipation and the risk of large artery atherosclerotic stroke (OR = 1.5, 95% CI = 1.07–2.104, *p* = 0.019). Conversely, other methods suggested no such association (Figure 4). However, our primary focus remained on the IVW results. No statistically significant association was observed between constipation and stroke or any of its subtypes (all *p* > 0.05), with the results presented in Figure 4. Leave-one-out analyses of these results described above are presented in Supplementary Figure S1, with no distinct strong SNP effects identified. Scatter plots are presented in Supplementary Figure S2. Forest plots are presented in Supplementary Figure S3, indicating the size of the MR effect of constipation on stroke and its subtypes.

#### 3.2.3. Sensitivity analysis

The heterogeneity detected in some outcomes does not invalidate MR results (Table 3). This is due to the employment of the random-effects IVW used in this study, which effectively mitigates pooled heterogeneity. Additionally, our analysis of funnel plots revealed that the effect-size variations around the point estimates were generally symmetric, indicating an absence of horizontal pleiotropy in the analysis (Supplementary Figure S4). In the MR-PRESSO global test and MR-Egger intercept test, all *p*-values exceeded 0.05, indicating the absence of horizontal pleiotropy in the analysis. The MR Steiger test confirmed the correctness of the direction, yet it yielded *p*-values above 0.05, suggesting the possibility of reverse causality. Consequently, an MR study with stroke and its subtypes as exposure and constipation as the outcome was conducted to assess causality. However, our analysis using Cochran’s Q test revealed heterogeneity between constipation and LS (Q = 23.59, *p* = 0.01) and between constipation and SVS (Q = 27.68, *p* = 0.02).

**Table 3:** Sensitivity analysis of the causal relationship between constipation and the risk of stroke and its subtypes.

Outcome	Cochran’s Q test Q-value	Value of *p*	MR-PRESSO Value of *p*	MR-Egger Intercept	Value of *p*	MR Steiger test Value of *p*
Stroke	13.62	0.48	0.41	−0.002	0.90	0.91
Ischemic stroke	17.53	0.18	0.17	−4.3e-06	0.99	0.91
Lacunar stroke	23.59	0.01	0.06	−0.000675	0.98	0.94
Cardioembolic stroke	14.19	0.36	0.29	0.01	0.65	0.91
Small-vessel stroke	27.68	0.02	0.06	−0.02	0.73	0.93
Large-artery atherosclerotic stroke	11.34	0.66	0.74	0.02	0.43	0.91

MR, Mendelian randomization; MR-PRESSO, MR Pleiotropy RESidual Sum and Outlier.

#### 3.2.4. Selection of genetic IVs for MR (stroke to constipation)

Seven SNPs associated with stroke, seven associated with IS, three associated with LS, 36 associated with CS, 32 associated with SVS, and 34 associated with LAS were obtained. These SNPs satisfied the currently accepted genome-wide significance criteria (*p* < 5 × 10^−6^, *R^2^* < 0.001, kb = 10,000) for exposure (Supplementary Table S8). F-statistics were calculated to assess the robustness of these SNPs, and all demonstrated substantial strength (all F-statistics > 10; Supplementary Table S8). Following the exclusion of outliers identified by MR-PRESSO, the SNPs that remained, after eliminating ambiguous and palindromic SNPs, were included as IVs. Details of the IVs for each stroke type and its associated subtype concerning constipation are presented in Supplementary Tables S9–S13.

#### 3.2.5. Causal effects of stroke and its subtypes on constipation

**Figure 5:**
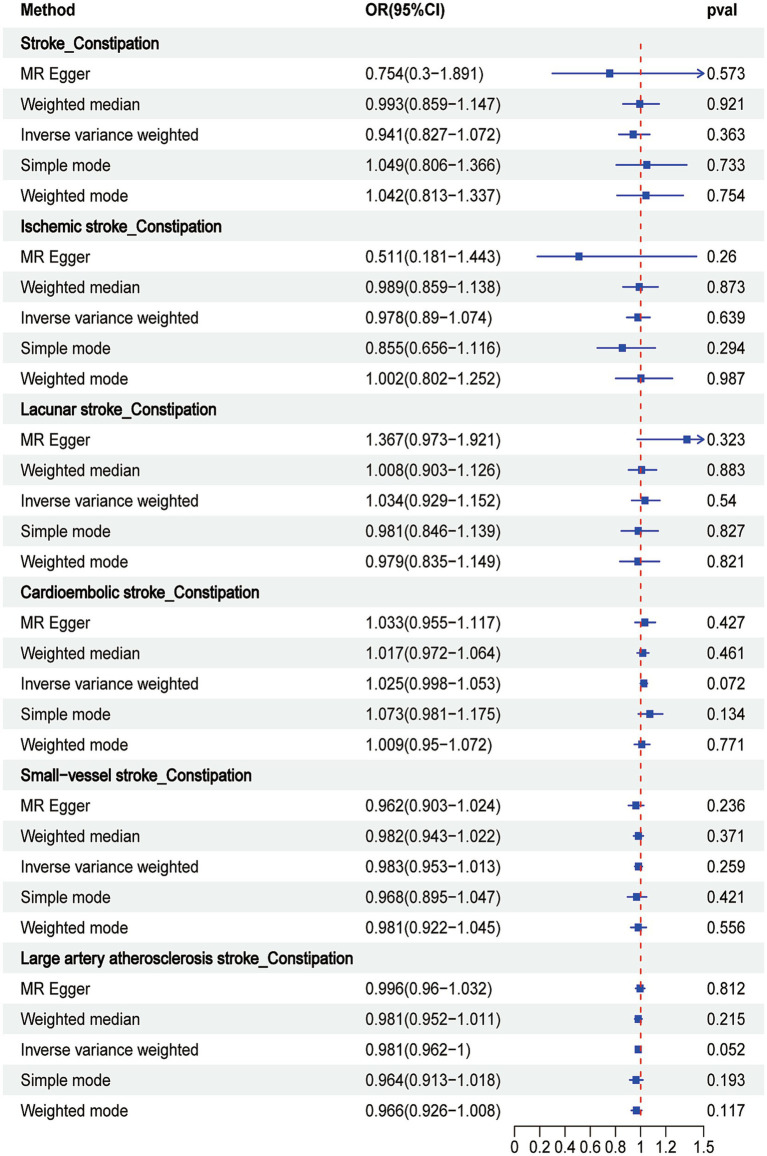
Associations of genetically predicted stroke and its subtypes with constipation by Mendelian randomization analysis.

The IVW analysis revealed no significant associations between constipation and either stroke or its subtypes. The following associations were observed: stroke and constipation (OR = 0.941, 95% CI = 0.827–1.072; *p* = 0.363), IS and constipation (OR = 0.978, 95% CI = 0.890–1.074; *p* = 0.639), LS and constipation (OR = 1.034, 95% CI = 0.929–1.152; *p* = 0.540), CS and constipation (OR = 1.025, 95% CI = 0.998–1.053; *p* = 0.072), SVS and constipation (OR = 0.983, 95% CI = 0.953–1.013; *p* = 0.259), and LAS and constipation (OR = 0.981, 95% CI = 0.962–1.000; *p* = 0.052). No significant correlations were observed with the employment of other methods (Figure 5). Leave-one-out analyses of the above-mentioned results are presented in Supplementary Figure S5, where no pronounced effects of SNPs were observed. Scatter plots are presented in Supplementary Figure S6, while forest plots are presented in Supplementary Figure S7, indicating the magnitude of the MR effect of constipation on stroke and its subtypes.

#### 3.2.6. Sensitivity analysis

Cochran’s Q test, MR-PRESSO global test, MR Steiger test, and MR-Egger intercept were performed to assess the robustness of the results (Table 4). The MR-PRESSO global test and MR-Egger intercept test produced *p*-values > 0.05, indicating the absence of horizontal pleiotropy in the analysis. However, Cochran’s Q test revealed heterogeneity between IS and constipation (Q = 14.25, *p* = 0.03) and cardiac stroke and constipation (Q = 54.23, *p* = 0.02). However, the heterogeneity observed in certain outcomes does not undermine the MR analysis. This is due to the use of random-effects IVW used in our study, which effectively mitigates pooled heterogeneity. Additionally, the funnel plot analysis indicated that the effect-size variations around the point estimates were generally symmetrical, further supporting the conclusion that horizontal pleiotropy was not apparent (Supplementary Figure S8).

**Table 4:** Sensitivity analysis of the causal relationship between stroke and its subtypes and the risk of constipation.

Outcome	Cochran’s Q test		MR-PRESSO	MR-Egger		MR Steiger test
	Q-value	Value of *p*	Value of *p*	Intercept	*p-*value	*p-*value
Stroke-constipation	9.21	0.16	0.20	0.01	0.65	-
Ischemic stroke-constipation	14.25	0.03	0.06	0.05	0.27	-
Lacunar stroke-constipation	3.51	0.17	0.12	−0.04	0.35	-
Cardioembolic stroke-constipation	54.23	0.02	0.19	−0.001	0.84	-
Small-vessel stroke-constipation	39.61	0.11	0.19	0.006	0.45	-
Large-artery atherosclerotic stroke-constipation	34.21	0.41	0.39	−0.005	0.34	-

MR, Mendelian randomization; MR-PRESSO, MR Pleiotropy RESidual Sum and Outlier.

## 4. Discussion

Upon comparing the clinical characteristics of patients with stroke with those of non-stroke patients, several risk factors for stroke emerged, including older age, non-Hispanic white ethnicity, marital status, higher education levels, and the presence of diabetes or hypertension. Subsequently, an analysis was conducted to assess the relationship between constipation and stroke. After adjusting for gender, age, race, and education, as well as confounding factors such as marital status, BMI, PIR, hypertension and diabetes, or smoking status and alcohol consumption, a significant association was observed between constipation and stroke. Regarding the MR analysis involving constipation and stroke and its subtypes, it was observed that constipation was associated with an increased risk of LAS in IVW method. IVW method generally have greater statistical power than other MR methods and is used as the primary method for identifying potentially significant results (Dong et al., 2023; Jia et al., 2023). We also performed a sensitivity analysis to ensure that the IVW estimates were robust. Finally, the MR analysis of constipation in stroke and its subtypes (IS, LS, LAS, SVS, and CES) revealed that neither stroke nor its subtypes were associated with constipation.

Chronic constipation, a prevalent condition, significantly affects a patient’s quality of life (Camilleri et al., 2017). Currently, the potential relationship between constipation and cardiovascular risk has been unexplored. While a limited number of studies have examined the association between constipation and stroke, the results across most studies have been inconsistent. In the past decade, several observational studies have investigated the association between chronic constipation and stroke, as well as its subtypes, yielding contradictory findings (Kubota et al., 2016; Ma et al., 2016). For instance, a cohort study suggested that severe constipation was associated with a 1.2-fold increased risk of atherosclerotic cardiovascular disease in postmenopausal women (Salmoirago-Blotcher et al., 2011). Similarly, a veteran cohort reported a 1.1-fold increased risk of coronary heart disease and a 1.2-fold increased risk of IS (Sumida et al., 2019). Consistently, a population-based matched cohort study in Denmark identified a higher risk of stroke in individuals with constipation compared to those without constipation (Sundbøll et al., 2020). It is noteworthy that the above studies did not account for confounding factors. For instance, after adjusting for potential confounding variables, Kubota et al. reported that constipation increased the risk of coronary heart disease and stroke without statistical significance (Kubota et al., 2016). Moreover, a recent MR analysis failed to provide evidence of a causal relationship between constipation and stroke or its subtypes (Dong et al., 2023). In our study, we first used logistic regression to determine the association between constipation and stroke in a cross-sectional study, and then conducted MR analysis. We enrolled more constipation participants than that study, which may explain why we found a causal relationship between constipation and LAS. Also, further research is warranted to elucidate whether bias or confounding factors influenced the outcomes of previous observational studies.

Colonic sensorimotor disorders and pelvic floor dysfunction are the main causes of constipation. Other factors such as reduced caloric intake, disruption of the microbiome, anatomical problems, or medications may also play a role in constipation (Bharucha and Lacy, 2020). The possible mechanisms by which constipation causes stroke remain inadequately characterized. It is hypothesized that the mechanisms linking constipation to increased cardiovascular risk are multifactorial and might vary according to the type of outcome. However, constipation and stroke share numerous risk factors, including age (Drossman et al., 1993), diabetes (Camilleri et al., 2017), depression, physical inactivity (Camilleri et al., 2017), and low dietary fiber intake (Dukas et al., 2003).

Decreased gut motility observed in patients with constipation might contribute to microbiome dysbiosis (Musso et al., 2011). Recent research has consistently highlighted the pivotal role of the gut microbiome in the development and progression of atherosclerosis and cardiovascular disease in large clinical cohorts. The increased transit time in patients with constipation might further facilitate the translocation of proinflammatory cytokines from gut bacteria, resulting in an inflammatory response and enhanced oxidative stress (Musso et al., 2011). Hence, the underlying chronic systemic inflammatory state observed in patients with constipation could be a risk factor for stroke (Grassi et al., 2015). This mechanism could elucidate the association between constipation and distant cardiovascular disease, providing a basis for our observed increased risk of IS, myocardial infarction, and peripheral artery disease. A recent study (Kop et al., 2010) has established a positive association between constipation and various gastrointestinal cancers. This might partly account for findings that the risk of splanchnic vein thrombosis is considerably higher compared to that of deep vein thrombosis and pulmonary embolism.

Our study has several strengths. The primary advantage is that an initial correlation analysis was performed, which established the association between constipation and stroke incidence. Based on this, a two-sample bidirectional MR analysis was applied to evaluate the independent causal effect of constipation on stroke and its subtypes, which was not confounded by reverse causality or residual confounding. Furthermore, the most significant GWAS with a large sample size was employed, ensuring our MR analysis was adequately powered.

Our study has certain limitations. One inherent challenge in all MR studies is the potential presence of directional pleiotropy, which cannot be entirely eliminated. However, our findings, coupled with the results of sensitivity analyses, did not indicate the presence of pleiotropy, lending robustness to our causal effect estimates. Furthermore, not all factors contributing to stroke risk have been exhaustively enumerated, for example, dietary habits, other surgical history, the presence of coronary heart disease, and mental health status, among others, could potentially play a role in stroke risk but have not been thoroughly explored in this study. Another limitation pertains to the population scope of our study, which was limited to individuals of European descent. Consequently, the generalizability of our findings to other ethnic groups might be constrained, as the incidence of constipation and stroke could differ in East Asian populations. However, given the European ancestry of our study cohort, it is unlikely that demographic stratification bias influenced our results.

## 5. Conclusion

Our study, employing a cross-sectional approach, demonstrated a positive association between constipation and stroke. Moreover, a two-sample bidirectional MR analysis confirmed the causal association between constipation and LAS but not between stoke or its other subtypes (IS, LS, SVS, and CES). These findings might pave the way for novel approaches to constipation management in the future.

## Data availability statement

The datasets presented in this study can be found in online repositories. The names of the repository/repositories and accession number(s) can be found in the article/Supplementary material.

## Ethics statement

The studies involving humans were approved by Health Statistics Research Ethics Review Board. The studies were conducted in accordance with the local legislation and institutional requirements. Written informed consent for participation was not required from the participants or the participants’ legal guardians/next of kin in accordance with the national legislation and institutional requirements.

## Author contributions

WD: Writing – review & editing, Data curation, Formal analysis, Writing – original draft. SY: Formal analysis, Writing – review & editing, Visualization. HZ: Data curation, Writing – original draft. YW: Writing – review & editing. YC: Writing – review & editing. HM: Writing – review & editing. HT: Writing – review & editing, Supervision. AH: Supervision, Conceptualization, Methodology, Writing – review & editing.

## Footnotes

^1^https://www.cdc.gov/nchs/nhanes/irba98.htm

^2^https://www.cdc.gov/nchs/data_access/restrictions.htm

^3^https://www.finngen.fi/en

^4^https://gwas.mrcieu.ac.uk/
