# Supplementary material for: The association between constipation and stroke based on the NHANES and Mendelian randomization study
Source: Front Neurosci. 2023 Nov 7;17:1276032. doi: 10.3389/fnins.2023.1276032 (PMC10661951; doi:10.3389/fnins.2023.1276032)
Supplement: Supplementary file 1 [file Data_Sheet_1.DOCX]

**Supplementary Figure Legends**

**Supplementary Figure 1**

Leave-one-out sensitivity analysis of the association between a genetic predisposition to constipation and risk of stroke or other stroke subtypes. A: Stroke; B: Ischemic stroke; C: Lacunar stroke; D: Cardioembolic stroke; E: Small-vessel stroke; F: Large-artery atherosclerotic stroke. The dot and bar indicate the estimates and 95% confidence interval when the specific single nucleotide polymorphism is removed.

**Supplementary Figure 2**

Scatter plot of the association between a genetic predisposition to constipation and risk of stroke or other stroke subtypes. A: Stroke; B: Ischemic stroke; C: Lacunar stroke; D: Cardioembolic stroke; E: Small-vessel stroke; F: Large-artery atherosclerotic stroke. Each black dot indicates a SNP, plotted by the estimate of SNP on stroke or other stroke subtypes and the estimate of SNP on the risk of constipation with standard error bars. The slopes of the lines correspond to causal estimates using each of the different methods. SNP: single nucleotide polymorphism.

**Supplementary Figure 3**

Forest plot of the association between a genetic predisposition to constipation and risk of stroke or other stroke subtypes. A: Stroke; B: Ischemic stroke; C: Lacunar stroke; D: Cardioembolic stroke; E: Small-vessel stroke; F: Large-artery atherosclerotic stroke. The dot and bar indicate the causal estimate of stroke or other stroke subtypes on risks of constipation.

**Supplementary Figure 4**

Funnel plot of the association between a genetic predisposition to constipation and risk of stroke or other stroke subtypes. A: Stroke; B: Ischemic stroke; C: Lacunar stroke; D: Cardioembolic stroke; E: Small-vessel stroke; F: Large-artery atherosclerotic stroke. Each black dot indicates a single nucleotide polymorphism.

**Supplementary Figure 5**

Leave-one-out sensitivity analysis of the association between a genetic predisposition to stroke and its subtypes and risk of constipation. A: Stroke; B: Ischemic stroke; C: Lacunar stroke; D: Cardioembolic stroke; E: Small-vessel stroke; F: Large-artery atherosclerotic stroke. The dot and bar indicate the estimates and 95% confidence interval when the specific single nucleotide polymorphism is removed.

**Supplementary Figure 6**

Scatter plot of the association between a genetic predisposition to stroke and its subtypes and risk of constipation. A: Stroke; B: Ischemic stroke; C: Lacunar stroke; D: Cardioembolic stroke; E: Small-vessel stroke; F: Large-artery atherosclerotic stroke. Each black dot indicates a SNP, plotted by the estimate of SNP on stroke and its subtypes and the estimate of SNP on the risk of constipation with standard error bars. The slopes of the lines correspond to causal estimates using each of the different methods. SNP: single nucleotide polymorphism.

**Supplementary Figure 7**

Forest plot of the association between a genetic predisposition to stroke and its subtypes and risk of constipation. A: Stroke; B: Ischemic stroke; C: Lacunar stroke; D: Cardioembolic stroke; E: Small-vessel stroke; F: Large-artery atherosclerotic stroke. The dot and bar indicate the causal estimate of stroke and its subtypes on the risk of constipation.

**Supplementary Figure 8**

Funnel plot of the association between a genetic predisposition to stroke and its subtypes and risk of constipation. A: Stroke; B: Ischemic stroke; C: Lacunar stroke; D: Cardioembolic stroke; E: Small-vessel stroke; F: Large-artery atherosclerotic stroke. Each black dot indicates a single nucleotide polymorphism.
