# Supplementary figures and images for: The association between constipation and stroke based on the NHANES and Mendelian randomization study
Source: Front Neurosci. 2023 Nov 7;17:1276032. doi: 10.3389/fnins.2023.1276032 (PMC10661951; doi:10.3389/fnins.2023.1276032)

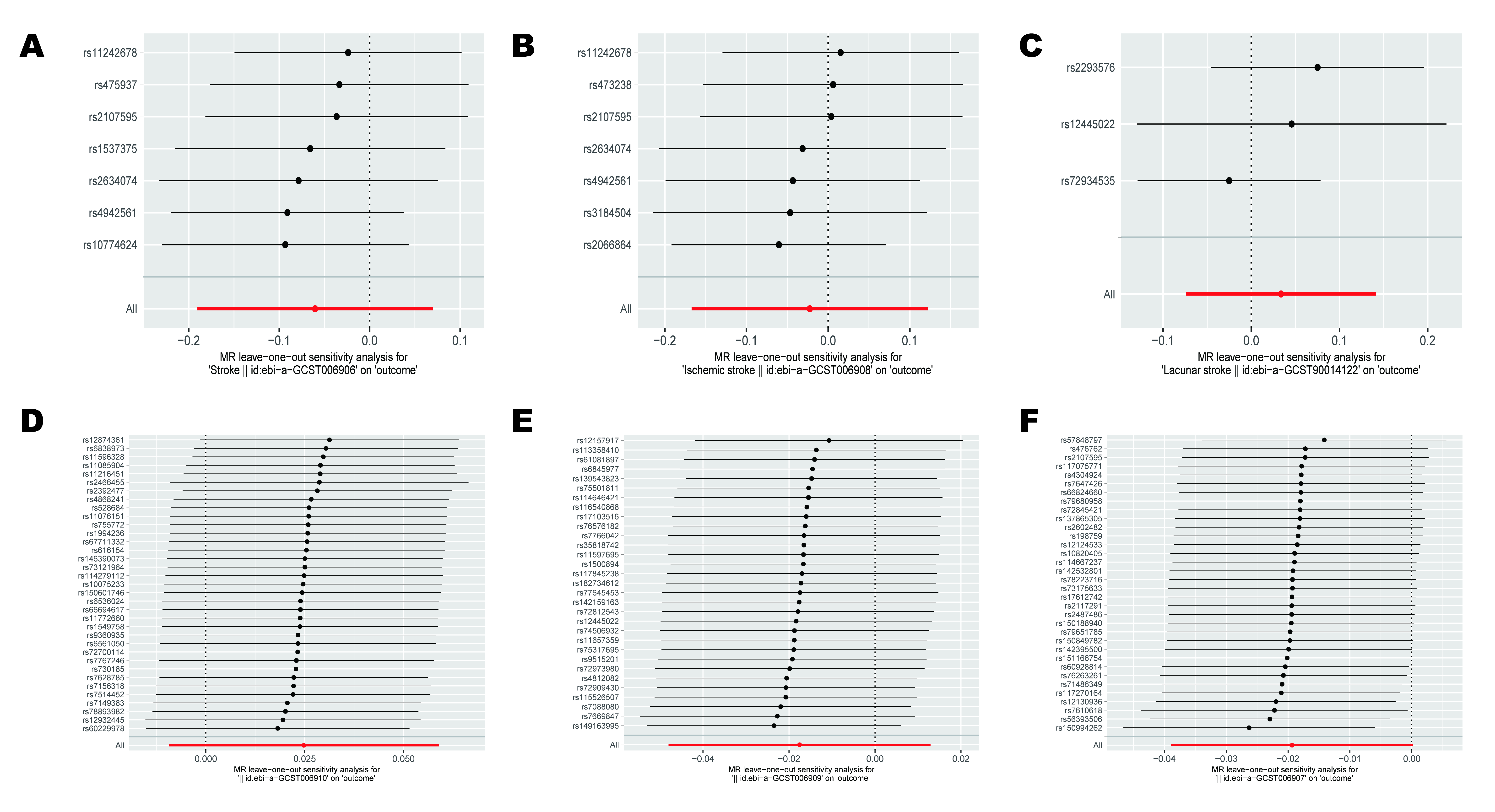

Supplement: Supplementary file 6 [file Image_5.TIF]

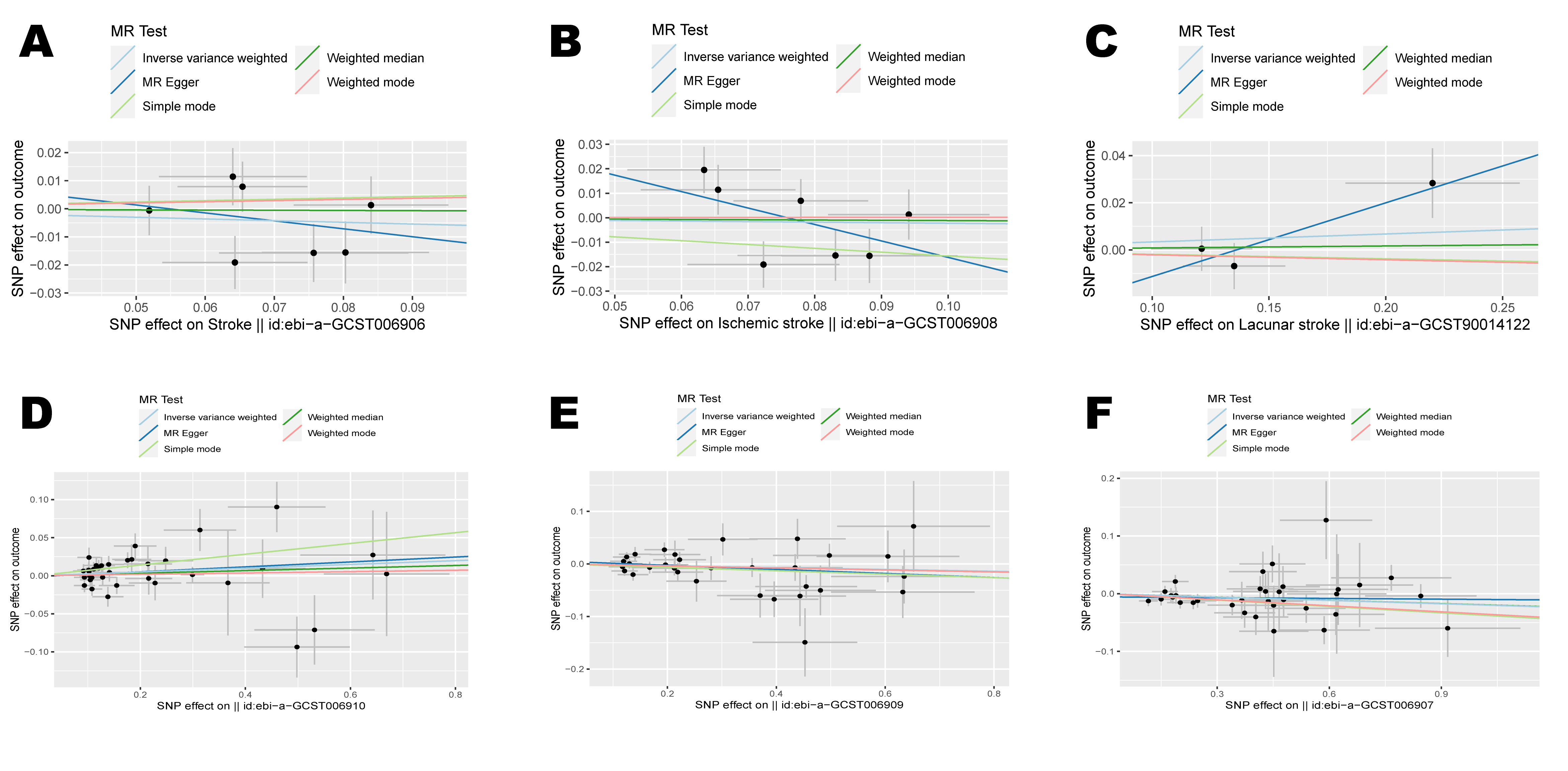

Supplement: Supplementary file 7 [file Image_6.TIF]

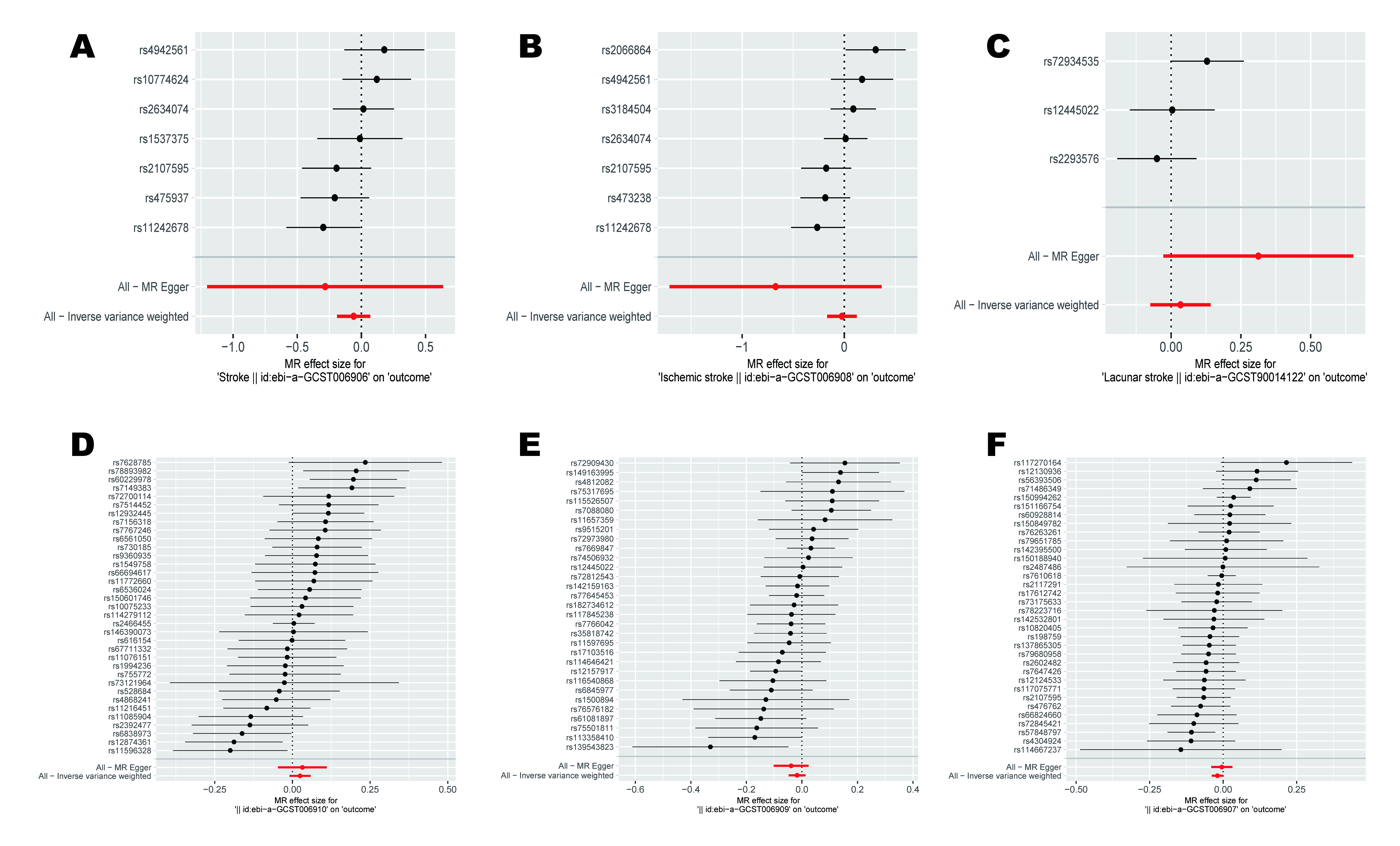

Supplement: Supplementary file 8 [file Image_7.TIF]

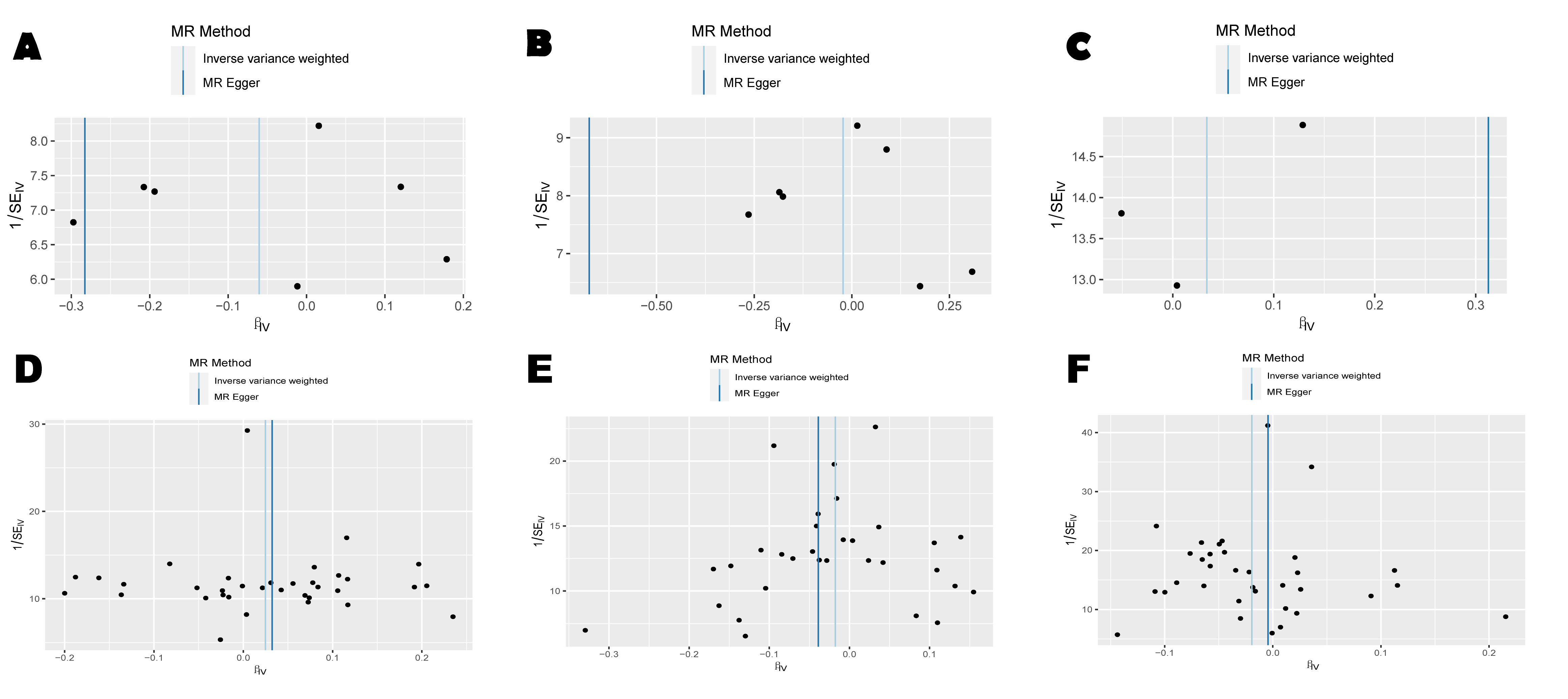

Supplement: Supplementary file 9 [file Image_8.TIF]
